# Ubiquitin is associated with the survival of ectopic stromal cells in endometriosis

**DOI:** 10.1186/1477-7827-2-69

**Published:** 2004-09-25

**Authors:** Romina S Ilad, Steven D Fleming, Catherine R Bebington, Christopher R Murphy

**Affiliations:** 1 Department of Obstetrics and Gynaecology, University of Sydney, New South Wales, Australia; 2 Department of Reproductive Medicine, Westmead Hospital, New South Wales, Australia; 3 Department of Anatomy and Histology, University of Sydney, New South Wales, Australia

## Abstract

**Background:**

Endometriosis is a condition that affects women of reproductive age, where the glandular and/or stromal tissues from the eutopic endometrium implant in ectopic locations. It is well established that the survival of ectopic implants is due to lower levels of apoptosis, but no consensus exists as to which pathway/s this is mediated by. The ubiquitin protein shares a similar sequence homology to an anti-apoptotic protein called BAG-1 and is expressed in the normal endometrium. Currently, no studies have been conducted to determine ubiquitin expression and its possible anti-apoptotic effects in endometriosis.

**Methods:**

Archived endometrial tissues from endometriosis patients and women undergoing laparoscopic diagnosis (controls) from January 2000 to July 2003 at Westmead Hospital were examined, where 14 cases of endometriosis and 55 controls were included in the study.

**Results:**

Both the ubiquitin protein and apoptosis were expressed in both glandular and stromal cells throughout the menstrual cycle of the eutopic endometrium, in which ubiquitin exhibited a cyclic expression, reaching a peak in late proliferative phase. In contrast, ubiquitin was predominantly expressed in cells of stromal origin in endometriosis, was no longer regulated by a cyclic pattern and was associated with an aberrant level of cell survival.

**Conclusions:**

For the first time, this study shows that ubiquitin is expressed in endometriotic cells and may contribute to a reduced sensitivity of ectopic endometrial tissue to apoptosis. These findings also suggest that stromal cells contribute differentially to the development of ectopic endometrial tissue.

## Background

Endometriosis is a condition that affects 10% of women of reproductive age [1]. In the condition, endometrial glands and/or stroma from the eutopic endometrium lodge and survive at ectopic sites such as the fallopian tubes, ovaries and peritoneal cavity. The most accepted theory as to how these cells migrate to ectopic areas is Sampson's retrograde menstrual transport theory, where endometrial cells are shed through the fallopian tubes [2]. However, what is not known is how these foreign cells continue to survive in their new ectopic location.

Ubiquitin is a 76 amino acid protein [3] that is involved in the degradation of short lived, regulatory or misfolded proteins, thus maintaining cellular homeostasis [4]. Ubiquitin tags these proteins to be taken to the proteasome and in some instances also to the lysosomic machinery to prevent damage to cells. This is the most cited function of ubiquitin, although ubiquitin also shares a very similar sequence homology to the B-cell lymphoma athanogene 1 (BAG-1), which has known anti-apoptotic properties. To our knowledge, the hypothesis that ubiquitin may mediate anti-apoptosis within the endometrium has never been examined and consequently has not been investigated in the area of ectopic endometrial cell survival in endometriosis [5].

Accumulating evidence suggests that dysregulated apoptotic machinery in endometriosis has a role in its pathogenesis, but no consensus exists as to which pathway/s this is mediated by [6]. Transformed ectopic endometrial cells are able to survive due to a number of factors, such as down regulation of soluble Fas concentrations and a lack of cell-surface Fas expression on T-cells [7].

Therefore, this study was undertaken to determine the expression of ubiquitin within endometriosis during the menstrual cycle and to establish whether it is related to a decrease in the incidence of apoptosis in this tissue, thus potentially promoting ectopic endometrial cell survival.

## Methods

### Tissues

Approval to conduct this study was granted by the Human Research Ethics Committee of the Western Sydney Area Health Service. Archived formalin fixed, paraffin embedded tissues from 14 women with endometriosis and 55 women undergoing laparoscopy for non-endometrial pathologies such as leiomyomata and benign ovarian cyst were studied. Endometriotic implants (n = 20) and endometrial tissues (n = 59) were obtained from the women above, respectively. All tissues were observed from the Department of Pathology at Westmead Hospital between January 2000 and July 2003. Mean ages for endometriosis and control groups were 42.5 ± 2.69 and 36.4 ± 1.17 years, respectively. Women with histological evidence of malignancy, necrosis, active inflammation or hormonal treatment were excluded from the study. Control subjects had normal, regular menstrual cycles and endometrial samples were collected from the proliferative and secretory phases of the cycle.

### Sectioning

Sections (4 μm) of endometriosis and control endometrial tissue blocks were cut on a microtome (Microtome, Stauffenberg, Germany), placed on superfrost slides (Menzel-Glaser, Braunscheig, Germany) and air-dried at room temperature for 24 hours.

### Localisation of ubiquitin

De-waxing was performed with two changes of histolene and absolute ethanol through a single change of 95% (v/v) and 70% (v/v) ethanol and a final change of tap water. All incubations were of 5 min duration. Following the suppression of endogenous peroxidase activity for 10 min with a blocking agent (DAKO Pty Ltd, Botany, Australia) sections were incubated with normal goat serum for 60 min to prevent non-specific binding. A polyclonal rabbit anti-ubiquitin primary antibody (DAKO Pty Ltd, Botany, Australia) prepared at a titre of 1:100 was added for a further 60 min at 37°C. Slides were then washed three times in tris-buffered saline (TBS: 50 mM Tris HCl, 150 mM NaCl, pH 7.5; 5 min) before the biotinylated goat anti-rabbit IgG secondary antibody (DAKO Pty Ltd, Botany, Australia) prepared at 1:200 was applied (30 min). A horseradish peroxidase avidin biotinylated complex kit (HRP-ABC) and diaminobenzidine (DAB) were used according to the manufacturer's instructions (DAKO Pty Ltd, Botany, Australia) to detect the secondary antibody. All washes used TBS except after DAB administration, where water was used. Sections were counterstained with haematoxylin and eosin (H&E) and cover slips were attached using a DAKO aqueous based mounting medium. Negative controls were obtained through the omission of the primary antibody to ubiquitin (that showed the absence of specific staining). Ubiquitin has increased expression in the normal endometrium in both the late proliferative and late secretory phase. In this study, late secretory phase tissue was used as a positive control [8].

Currently, no single assay exists that detects apoptosis with high specificity and sensitivity [9]. Thus the TUNEL technique used in this study was used in conjunction with the classical method of H&E staining (which detects nuclear shape changes during the early stages of apoptosis) as positive strand break detection alone (TUNEL) may overestimate the true occurrence of cell death within a given cell population. A presence of DNA strand breaks, for example, may not correlate with nuclear segmentation or may be detected during the late lytic stage of apoptosis, where most cells are no longer viable.

### TUNEL labelling

Sections were deparaffinized and rehydrated with histolene and a graded series of alcohols (absolute, 95%, 90%, 80% and 70%) for 5 min each. This was followed by a 20 min incubation of sections with proteinase K [15 μg/ml in 10 mM Tris/HCl, pH 7.5] at room temperature. Sections were then rinsed twice in phosphate-buffered saline (PBS) and reacted with 50 μl of the TUNEL reaction mixture (Roche Diagnostics, Castle Hill, Australia) for 60 min in a dark, humidified chamber at room temperature. The sections were then rinsed three times in PBS and incubated for a further 30 min with 50 μl of the Converter-POD (Roche Diagnostics, Castle Hill, Australia) followed by 10 min with DAB. This procedure ensures the detection of TUNEL labelled cells. Finally, sections are counterstained and mounted as described for ubiquitin staining. For positive controls, sections were treated with DNAse 1 to induce DNA strand breaks or peroxidase blocking solution was excluded. Negative controls were achieved by omitting terminal deoxynucleotidyl transferase (TdT; Roche Diagnostics, Castle Hill, Australia).

### H&E stains

The Department of Pathology, Westmead Hospital, kindly provided the H&E slides used for this study. The basis for this procedure is to identify cells with cell blebbing, nuclear condensation and cell shrinkage, which are characteristic features of apoptosis.

### Scoring of ubiquitin sections

The ubiquitin staining in the normal and endometriotic tissue was calculated using a semi-quantitative method to determine the average intensity scores of the protein in the nucleus of glands and the stroma using an Optimas Image Analysis program (Silver Spring, USA). Five randomly selected fields were viewed and evaluated with a grading of 0, 1, 2 or 3 (negative, weak, moderate, strong) according to a scale created by Watanabe and colleagues [10].

### Scoring of TUNEL sections

TUNEL apoptotic cell numbers were determined by counting darkly labelled cells in five randomly selected fields at X400 and expressed as the apoptotic cell mean/field according to Meresman and colleagues [11].

### Scoring of H&E sections

Ten randomly chosen fields at X600 magnification were used to determine the number of apoptotic stromal and glandular cells according to a modified grading scale used by Meresman and colleagues [12]: (-) < 3 apoptotic cells/field; (+) >3 apoptotic cells/field.

This modified grading system was used to make statistical analysis more accurate, as there were only a few samples that exhibited > 8 apoptotic cells/field. Also, apoptotic cells were identified by their characteristic morphological features in H&E-stained late secretory endometrial sections. These included cell shrinkage and chromatin margination or chromatin condensation with formation of apoptotic bodies [12].

Apoptotic bodies were identified by the presence of one, or several of the following features [13,14]:

1) Single round mass of condensed strongly eosinophilic cytoplasm with a clump of strongly basophilic, homogenous chromatin.

2) Cytoplasm with more than one piece of condensed chromatin.

3) Condensed chromatin fragments without cytoplasm.

### Statistical analysis

All data is expressed as mean ± SEM. Comparisons between experimental groups were performed using the independent t-test and Mann-Whitney U test for parametric and non-parametric analysis, respectively. A P-value less than 0.05 was considered a significant difference between groups.

## Results

### Immunohistochemistry of ubiquitin in the control endometrium and endometriotic implants

**Figure 1:**
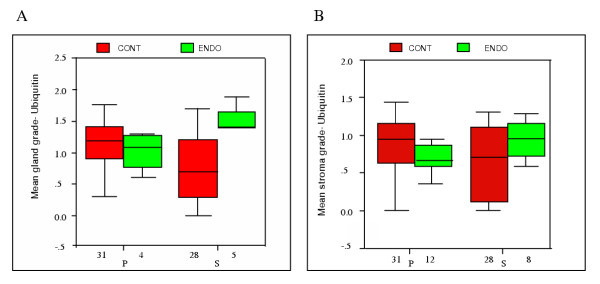
A. **Ubiquitin grades for glandular cells of control endometrium and endometriotic implants during the menstrual cycle**. Bars represent mean ± SEM; p < 0.05. P = proliferative and S = secretory. B **Ubiquitin grades for stromal cells of control endometrium and endometriotic implants during the menstrual cycle. **Bars represent mean ± SEM; p < 0.05. P = proliferative and S = secretory.

**Figure 3:**
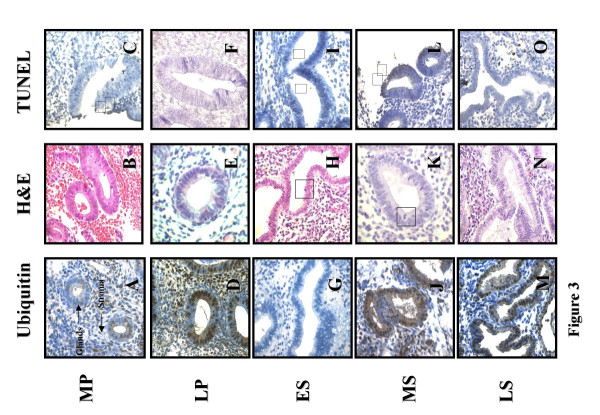
**Immunohistochemical staining of control endometrium. **Panels A, D,G,J and M are ubiquitin stained sections where brown cells are ubiquitin labelled (X400). Panels B, E, H, K and N are H&E stained sections where □ are apoptotic cells (X600). Panels C, F, I, L and O are TUNEL stained sections where ▼ are TUNEL positive cells.; *Menstrual cycle phases*: MP = mid proliferative; LP = late proliferative; ES = early secretory; MS = mid secretory and LS = late secretory.

**Figure 4:**
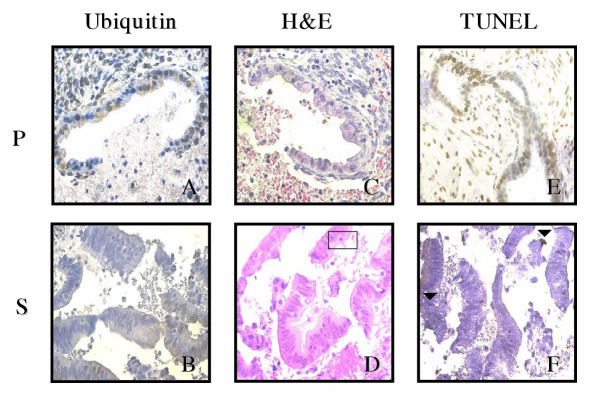
**Immunohistochemical staining of endometriotic implants. **Panels A and B are ubiquitin stained sections where brown cells are ubiquitin labelled (X400). Panels C and D are H&E stained sections where □ are apoptotic cells (X600). Panel E and F are TUNEL stained sections where ▼ are TUNEL positive cells. *Menstrual cycle phases*: P = proliferative and S = secretory.

Analysis of the effect of the menstrual cycle phase on glandular epithelial cell ubiquitin expression showed a greater level of ubiquitin during the proliferative phase than the secretory phase in controls (P = 0.032; Figure 1A, 3A,3D,3G,3J, and 3M) in contrast to ectopic glandular cells of endometriosis tissues where ubiquitin is higher during the secretory phase (P = 0.022; Figure 1A, 4A and 4B).

Furthermore, the effect of the menstrual cycle phase on stromal cell ubiquitin expression, showed a similar level of ubiquitin between the proliferative and secretory phase of controls (P > 0.05) in contrast to ectopic stromal cells of endometriosis tissues where ubiquitin is higher during the secretory phase (P = 0.020; Figure 1B and 4B).

### Detection of apoptotic cells in the control endometrium and endometriotic implants

**Figure 2:**
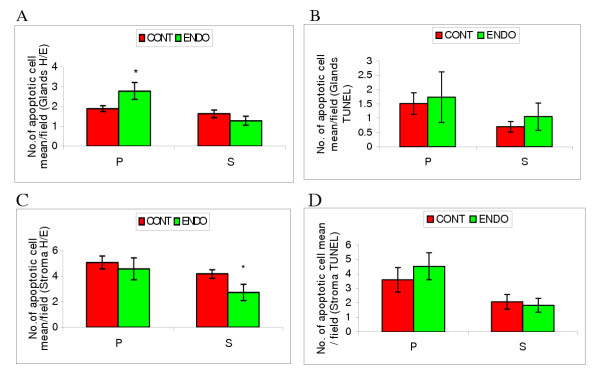
A. **Apoptotic grades for glandular cells of control endometrium and endometriotic implants during the menstrual cycle using H&E. **Data are expressed as the apoptotic cell mean/field. Bars represent mean ± SEM; *p < 0.05. *Menstrual cycle phases*: P = proliferative and S = secretory. B. **Apoptotic grades for glandular cells of control endometrium and endometriotic implants during the menstrual cycle using TUNEL **Data are expressed as the apoptotic cell mean/field. Bars represent mean ± SEM; *Menstrual cycle phases*: P = proliferative and S = secretory. C. **Apoptotic grades for stromal cells of control endometrium and endometriotic implants during the menstrual cycle using H&E. **Data are expressed as the apoptotic cell mean/field. Bars represent mean ± SEM; *p < 0.05. *Menstrual cycle phases*: P = proliferative and S = secretory. D. **Apoptotic grades for stromal cells of control endometrium and endometriotic implants during the menstrual cycle using TUNEL. **Data are expressed as the apoptotic cell mean/field. Bars represent mean ± SEM; *Menstrual cycle phases*: P = proliferative and S = secretory.

A significantly higher level of apoptosis was observed using H&E in ectopic glandular cells of patients with endometriosis in comparison to controls in the proliferative phase (2.79 ± 0.43 vs 1.90 ± 0.15 respectively; P = 0.04; Figure 2A, 3B,3E and 4C) whereas no significant difference was seen during the secretory phase (1.29 ± 0.23 vs 1.64 ± 0.19 respectively; P > 0.05; Figure 2A, 3H,3K,3N and 4D).

Similar levels of apoptosis were observed within proliferative phase ectopic stromal cells of patients with endometriosis and controls using the H&E method (4.57 ± 0.85 vs 5.08 ± 0.50 respectively; P > 0.05; Figure 2C, 3B,3E and 4C). In contrast, a considerably lower level of apoptosis was observed using H&E in secretory phase ectopic stromal cells of patients with endometriosis than in controls (2.73 ± 0.63 vs 4.17 ± 0.33 respectively; P = 0.03; Figure 2C, 3H,3K,3N and 4D).

Apoptotic cells are seen using the TUNEL technique (Figure 3C,3F,3I,3L,3O, 4E and 4F). However no statistically significant difference in levels of cell death were observed for both ectopic glands of endometriosis patients and controls in either the proliferative (1.73 ± 0.88 vs 1.51 ± 0.38 respectively; P > 0.05; Figure 2B, 3C,3F and 4E) or secretory phase (1.05 ± 0.48 vs 0.70 ± 0.19 respectively; P > 0.05; Figure 2B. 3I,3L,3O and 4F). Similar levels of apoptosis were also observed in ectopic stromal cells of endometriosis patients and controls using the TUNEL technique in both the proliferative (4.53 ± 0.93 vs 3.59 ± 0.85 respectively; P > 0.05; Figure 2D, 3C,3F and 4E) and secretory phase (1.83 ± 0.43 vs 2.08 ± 0.52 respectively; P > 0.05; Figure 2D, 3I,3L,3O and 4F).

## Discussion

Ubiquitin is implicated in the removal of short lived, regulatory or misfolded proteins but is also widely known to play a part in DNA repair and removal of virus budding [4]. The expression of ubiquitin during the proliferative phase of control tissues is likely to be modulated by increasing oestrogen since there is an increasing oestrogen level in response to FSH during folliculogenesis. Thus ubiquitin may play a role in supporting the developing endometrium should implantation occur, as its maximal expression correlates to day 14 of the menstrual cycle. In addition, Bebington and colleagues have previously shown an increase in ubiquitin levels during the late secretory phase as an indication that the protein may take part in the differentiation of the endometrium [8].

In this study, apoptosis was found to be present in endometriotic tissue with varying intensity. A previous study of endometriosis has also shown this result [15] but the possible biological relationship of ubiquitin to this condition has not previously been elucidated.

Our data shows that the expression of ubiquitin is increased during the secretory phase of the menstrual cycle in both glands (Figure 1A) and stroma (Figure 1B) in endometriosis tissues compared to controls. In addition, the level of apoptosis observed within the glands was greater during the proliferative phase (Figure 2A), in contrast with a significant decrease in apoptosis in ectopic stromal cells during the secretory phase (Figure 2C).

This study suggests that increased levels of ubiquitin within ectopic endometrial cells may allow their continued survival, through a yet to be established pathway. The up-regulation of ubiquitin in human endometriotic tissue may facilitate ectopic endometrial cell survival, particularly allowing those of stromal origin to grow, survive and evade T-cell mediated disposal. This finding is of particular interest because ubiquitin has primarily been attributed to the removal of aberrant proteins but seems, from our data, to be also associated with cell survival.

This duality in ubiquitin's role may be attributed to the type of lysine residue linkage that occurs during polyubiquitination. Studies by Deng and colleagues [16] have shown that if ubiquitin proteins are linked to each other through lysine 48, the target protein (in this case proteins on ectopic endometrial cells), will be directed to the proteasome for degradation. However, if the linkage occurs through lysine 63, the target protein associates with other proteins, aiding in its survival.

Our data is consistent with the hypothesis that ubiquitin has a protective effect on ectopic endometrial cells, particularly those of stromal origin, as shown by the increased level of ubiquitin with an associated decrease in apoptosis during the secretory phase. However, a different mechanism may apply to glandular endometriosis, where despite a greater ubiquitin expression during the secretory phase, no significant association was found with ectopic glandular cell survival. This suggests that an insufficient ubiquitin tagging during the proliferative phase may cause ectopic glandular cells to undergo apoptosis.

Other factors that may explain ectopic endometrial cell survival include the down-regulation of apoptotic receptors [15], failure of immune cells to recognise and eliminate ectopic cells [17] and an increase in cytokine levels and growth factors in the peritoneal fluid of women with endometriosis [18].

The significant increase in age of patients with endometriosis may have an unknown effect on the level of apoptosis as older women may have increased incidence of endometrial cell death due to increased longevity. However, this age difference is consistent with another study that also shows a higher age range in women with the condition [19].

Our results suggest that ubiquitin is important for endometrial function throughout the menstrual cycle where it may play an important role in the regeneration of the endometrium [5]. Also, a loss of ubiquitin regulation in the ectopic environment may have an important role in the pathogenesis of endometriosis. In this respect, it is interesting that ubiquitin has recently been shown to exert an immunomodulatory effect in other tissues [20][21]. Endometriotic implants may survive at ectopic locations due to a combination of factors, including protection from apoptosis and immune attack.

## Conclusions

In conclusion, this study demonstrates for the first time that ubiquitin is expressed in endometriotic cells. Furthermore, the up-regulation of ubiquitin expression may contribute preferentially to the development of these ectopic endometrial lesions due to their reduced sensitivity to apoptosis.

## Authors' contributions

RI was responsible for carrying out this study as part of her honours degree. CB provided RI with training in the techniques used. SF and CM were responsible for the conception and design of the study, research funding, and for supervision of RI's work. SF, CB and CM read and approved the final manuscript.
